# Estimation of Soil Organic Carbon Content in the Ebinur Lake Wetland, Xinjiang, China, Based on Multisource Remote Sensing Data and Ensemble Learning Algorithms

**DOI:** 10.3390/s22072685

**Published:** 2022-03-31

**Authors:** Boqiang Xie, Jianli Ding, Xiangyu Ge, Xiaohang Li, Lijing Han, Zheng Wang

**Affiliations:** 1 School of Geographical Sciences, Xinjiang University, Urumqi 830046, China; 1437136910@stu.xju.edu.cn (B.X.); gxy3s@stu.xju.edu.cn (X.G.); lxh_xju@stu.xju.edu.cn (X.L.); 107556519106@stu.xju.edu.cn (L.H.); wz_wangzheng@stu.xju.edu.cn (Z.W.); 2 Xinjiang Key Laboratory of Oasis Ecology, Xinjiang University, Urumqi 830046, China; 3 Key Laboratory of Smart City, Environment Modelling of Higher Education Institute, Xinjiang University, Urumqi 830046, China

**Keywords:** ensemble learning algorithms, Landsat 8, Sentinel-2A, Sentinel-1A, soil organic carbon, digital soil mapping

## Abstract

Soil organic carbon (SOC), as the largest carbon pool on the land surface, plays an important role in soil quality, ecological security and the global carbon cycle. Multisource remote sensing data-driven modeling strategies are not well understood for accurately mapping soil organic carbon. Here, we hypothesized that the Sentinel-2 Multispectral Sensor Instrument (MSI) data-driven modeling strategy produced superior outcomes compared to modeling based on Landsat 8 Operational Land Imager (OLI) data due to the finer spatial and spectral resolutions of the Sentinel-2A MSI data. To test this hypothesis, the Ebinur Lake wetland in Xinjiang was selected as the study area. In this study, SOC estimation was carried out using Sentinel-2A and Landsat 8 data, combining climatic variables, topographic factors, index variables and Sentinel-1A data to construct a common variable model for Sentinel-2A data and Landsat 8 data, and a full variable model for Sentinel-2A data, respectively. We utilized ensemble learning algorithms to assess the prediction performance of modeling strategies, including random forest (RF), gradient boosted decision tree (GBDT) and extreme gradient boosting (XGBoost) algorithms. The results show that: (1) The Sentinel-2A model outperformed the Landsat 8 model in the prediction of SOC contents, and the Sentinel-2A full variable model under the XGBoost algorithm achieved the best results R^2^ = 0.804, RMSE = 1.771, RPIQ = 2.687). (2) The full variable model of Sentinel-2A with the addition of the red-edge band and red-edge index improved R^2^ by 6% and 3.2% over the common variable Landsat 8 and Sentinel-2A models, respectively. (3) In the SOC mapping of the Ebinur Lake wetland, the areas with higher SOC content were mainly concentrated in the oasis, while the mountainous and lakeside areas had lower SOC contents. Our results provide a program to monitor the sustainability of terrestrial ecosystems through a satellite perspective.

## 1. Introduction

Soil is the largest reservoir of carbon in terrestrial ecosystems and soil organic carbon (SOC) plays an important role in the global carbon cycle and soil ecosystems [1,2,3]. SOC far exceeds the amount of carbon in the atmosphere and vegetation, with almost twice the amount of carbon in the atmosphere and three times that in plants [4]. Thus, small changes in the carbon contents of soils can alter the concentration of carbon dioxide in the atmosphere, leading to global warming [5]. Countries around the world are actively addressing this challenge through a range of carbon sequestration policies [6]. SOC plays a key role in maintaining ecosystem services and agricultural productivity by improving soil structure, enhancing water retention and maintaining nutrient availability [7]. Furthermore, the UN Sustainable Development Goals have identified SOC as a key indicator for estimating degraded land areas in the context of achieving land degradation neutrality and targets by 2030 [1,8]. Therefore, a timely understanding of the spatial distribution of soil organic carbon content is important for the terrestrial carbon cycle, future climate change monitoring and ecosystem restoration.

SOC measurement methods have traditionally utilized chemometric methods; however, the collection and processing of large numbers of samples and indoor experimental analysis are labor intensive and time consuming [9,10]. With the rapid development of remote sensing technology, remote sensing has become widely used for digital mapping of soil properties (DSM) [11,12,13]. DSM is an effective method to reduce the field sampling and experimental costs associated with monitoring, analyzing and managing SOC from spatial and temporal perspectives [14,15]. However, the spatial distribution of SOC is constrained by the spatial environment, and previous studies have demonstrated that variability in environmental variables can significantly affect the spatial distribution of SOC [16].

The response mechanism of SOC exists in the electromagnetic spectrum (e.g., visible, near-infrared, shortwave infrared and microwave). The absorption of radiant energy by the molecule causes the amplitude of the chemical bond vibrations to become larger, leading to stretching and bending of the N-H, O-H and C-H groups. The strongest correlations of these stretching and bending vibrations with SOC occur in the red, near-infrared and shortwave infrared bands [17,18]. However, due to the limitations caused by the spectral resolution of satellite data, the use of individual band response relationships with SOC does not yet satisfy the need for high accuracy. Spectral indices exploit the synergy of two or more bands to effectively mine spectral information. Therefore, some spectral indices with strong correlations with SOC (e.g., the soil-adjusted vegetation index (SAVI), normalized difference vegetation index (NDVI) and normalized burn ratio 2 (NBR2)) are used to estimate SOC [2,19]. Because Asa Gholizadeh [20] demonstrated that of the Sentinel-2 multispectral sensor information (MSI) data, band 4 and band 5 (red-edge bands and red-edge information) are most sensitive to SOC, we hypothesize that there may be potential to construct spectral indices from red-edge bands to estimate SOC with high accuracy. In addition, a complex subsurface (e.g., vegetation cover, soil roughness, topographic relief, etc.) affects the quality of optical imagery, and recent studies have introduced topographic data and synthetic aperture radar (SAR) data. This is because SAR data are not only independent of cloud and rain imagery, but can also capture soil–vegetation relationships to predict soil chemistry [21].

The estimation of soil properties using remote sensing is related to the spatial and spectral resolution of the image. The higher the spectral and spatial resolution, the better the estimation accuracy. This is because from a spatial perspective, a coarse spatial resolution contains more mixed pixels. From a spectral perspective, a higher spectral resolution can better represent the differences in reflectance spectra at the surface. Landsat 8, applied in past moderate-resolution satellite monitoring, has six bands in the VIS-NIR-SWIR range, but the low spatial resolution (30 m) of these images limits the accuracy of SOC predictions. Sentinel-2A has 10 bands in the VIS-NIR-SWIR range, which is four more red-edge spectra (Red Edges 1, 2, 3 and 4) than Landsat 8. However, the role of the red-edge spectra is usually unconsidered in the current SOC studies. This is because the Red band, near-infrared and shortwave infrared have a corresponding relationship with SOC, and finer spectral information will help to detect the relationship between the spectrum and the SOC. Therefore, SOC estimation in Sentinel-2A should not only take into account the improved SOC prediction results due to spatial resolution, but should also fully exploit the synergy of spectra, especially the combination of variables containing red-edge bands.

In terms of SOC prediction methods, early studies used linear regression models, including linear mixed effects models [22], multiple linear regression and geographically weighted regression models [23,24], to link spectral data to SOC. The advantage of such linear models is their simplicity of application and ease of interpretation. However, the relationship between SOC and variables is commonly complex and nonlinear [25]. The rapid rise of data mining and machine learning provides favorable conditions for establishing nonlinear relationships between soil properties and remotely sensed data. Cubist [26,27], support vector machines (SVM) [28,29], categorical regression trees (CART) [30,31], random forests (RF) [32,33], gradient boosted decision trees (GBDT) [34] and extreme gradient boosted decision trees (XGBoost) [35] are widely used. Among these methods, tree-based model algorithms have demonstrated good prediction results [36]. RF is an extension of CART, which can effectively control the risk of overfitting and has been shown to be superior in dealing with nonlinear relationships [37]. XGBoost, an improvement of the GBDT algorithm, can perform regression tasks quickly and accurately through an additive training strategy and parallel computing [38]. Most previous SOC mapping studies, while using indices derived from remote sensing data as valid variables for SOC prediction, lack the ability to explore the application of red-edge bands when using Sentinel-2A data. Therefore, it is necessary to explore the capability of red-edge indices in SOC prediction. In addition, the applicability of the excellent XGBoost algorithm in this study area is not yet known. The Ebinur Lake wetland is located in an arid and semiarid zone, where extreme climatic conditions lead to fragile ecosystems. To prevent land degradation and achieve sustainable development goals, accurate monitoring of SOC is an important mission, and there is currently a lack of high-precision spatial mapping of SOC in the region. We expect good prediction results from the red-edge index and multivariate modeling strategies with superior ensemble learning algorithms.

Most of the SOC predictions were executed using a single Sentinel-2A or Landsat 8 image. Relatively little is known about the comparison between multiple modeling strategies and the two sensors. We conducted the Sentinel-2A and Landsat 8 model input variables identical by controlling for variables so that the effect of spatial resolution on SOC prediction can be presented. The development of the Sentinel-2A red-edge variable also provides more insight into how much the red-edge variable improved the accuracy of SOC predictions. The final high accuracy SOC prediction and spatial distribution map of SOC were obtained. To better compare the effects of spatial resolution and Sentinel-2A red-edge bands and red-edge indices on SOC predictions, we designed three sets of models, two of which were based on Landsat 8 and Sentinel-2A data, respectively, using the same variables but differing in spatial resolution. We refer to these two sets of models as the common variable Landsat 8 and common variable Sentinel-2A models. The other group is based on the common variable Sentinel-2A with the addition of the red-edge variable, which we call the full variable Sentinel-2A model. These three groups of models are used to estimate SOC by three machine learning algorithms, XGBoost, GBDT and RF, respectively. The aim was to (1) compare the performances of Landsat 8 and Sentinel-2A data in predicting SOC; (2) explore the contribution of the red-edge band and red-edge-derived index variables of Sentinel-2A in the prediction of SOC; and (3) explore the SOC prediction performance of the three machine learning algorithms.

## 2. Materials and Methods

### 2.1. Study Area

The Ebinur Lake wetland is located in Northwest China (44°30′–45°09′ N, 82°36′–83°50′ E) and is the largest alkaline lake wetland in the Xinjiang Uygur Autonomous Region of China, with characteristics typical of an arid zone lake wetland. It is also the lowest waterlogged point on the southwestern boundary of the Junggar Basin in Xinjiang [39]. The area is surrounded by mountains on three sides, with Maili Mountain to the north, Borokhoro Mountain to the south and Alatau Mountain to the west of the study area. The Ebinur Lake wetland has a typical mid-temperate arid continental climate. The mean annual temperature (MAT) is 6–8 °C. The mean annual precipitation (MAP) is 100–1600 mm. Evaporation is about 1600 mm [40]. The study area is rich in soil types, with widespread Arenosols, Solonetz and Solonchaks, according to the World Reference Base (WRB) for Soil Resources [41,42]. In addition, land cover and land use contain a variety of land types, such as water bodies, vegetation, wetlands, deserts and saline soils [43].

### 2.2. Soil Data Source

**Figure 1:**
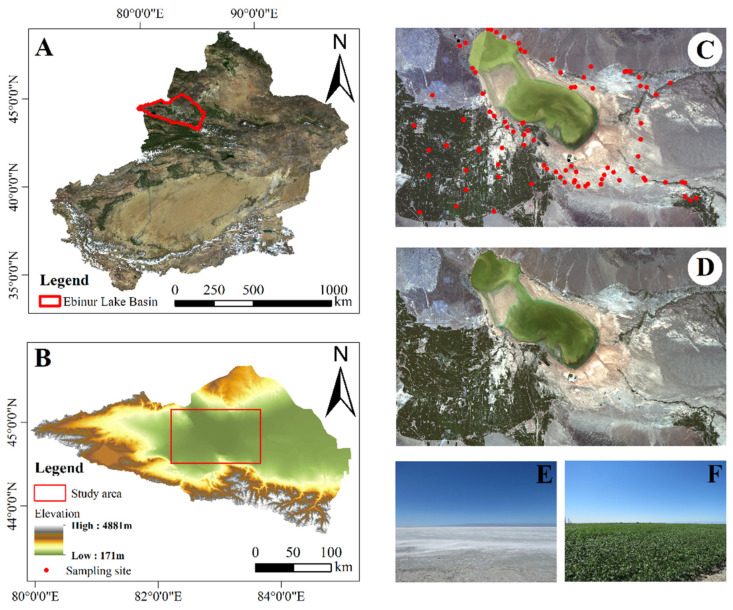
The study area is located in the Xinjiang Uyghur Autonomous Region within China: (**A**) Xinjiang Uyghur Autonomous Region within China; (**B**) The Ebinur Lake basin; (**C**) Sentinel-2A image; (**D**) Landsat 8 image; (**E**) landscape around the Ebinur Lake; (**F**) farmland landscape within the oasis. Images A, C and D were created using the Red, Green and Blue bands of remote sensing images.

Soil samples were obtained from field sampling at Ebinur Lake in July 2017. We set up a total of 95 sampling points in the study area, each consisting of a 10 m × 10 m sample square (Figure 1). Within each sample square, five soil surface samples (0–10 cm) were collected using a soil sampler following a five-point sampling method and mixed uniformly. The coordinates of the center of the sample square were recorded using a handheld GPS (UniStrong G120, error less than 5 m, Beijing UniStrong Science & Technology Corporation Limited, Beijing, China). Each soil sample was sealed and labeled in a soil collection bag and transported to the laboratory. The soil samples that were brought back to the laboratory underwent two steps: preprocessing and organic carbon testing. During soil preprocessing, the soil samples were first naturally air-dried; second, the air-dried soil samples were stripped of stones, weed roots and other impurities; and finally, the soil samples were ground and passed through a 0.149 mm grid sieve to obtain clean soil samples. In the organic carbon experiments, pretreated soil samples were pretreated with hydrochloric acid, mainly to eliminate the effect of soil salinity on the SOC prediction results. Finally, the soil organic carbon content was determined using the potassium dichromate method.

### 2.3. Environmental Variables

#### 2.3.1. Topographic Variables

Topographic analysis was carried out using Shuttle Radar Topography Mission (STRM) digital elevation data with a spatial resolution of 30 m, which were obtained from GEE (https://earthengine.google.com/, accessed on 21 October 2021). The projection of the digital elevation data was transformed in the GEE platform (WGS_1984_UTM_Zone_44N), cropped to the study area boundary and finally resampled to 10 m using the NEAREST method. We used digital elevation data at two resolutions (10 m and 30 m) and SAGA GIS software to calculate 15 terrain indices: the digital elevation model (DEM), analytical hillshading (AH), Slope, aspect, cross-sectional curvature (CSC), longitudinal curvature (LC), convergence index (ConI), closed depressions (CD), flow accumulation (FA), topographic wetness index (TWI), LS factor (LSF), channel network base level (CNBL), vertical distance to channel network (VDCN), valley depth (VD) and relative slope position (RSP).

#### 2.3.2. Remote Sensing Variables and Processing

The Landsat 8 Operational Land Imager (OLI) used for the study was launched by NASA in 2013. The data were acquired on 27 July 2017 from the USGS (http://glovis.usgs.gov/, accessed on 22 April 2021). The Landsat 8 data were radiometrically calibrated using the Radiometric Correction tool in ENVI5.3 software, and then atmospheric corrections were completed using the FLAASH atmospheric correction tool. Six bands were selected from the preprocessed Landsat 8 data to participate in the band calculation and modeling, as shown in Table 1. Sentinel-1A (Table 2) and Level 1-C Sentinel-2A data acquisition from ESA (https://scihub.copernicus.eu/dhus/#/home/, accessed on 21 October 2021) was accessed on 26 July 2017. Sentinel-1A provides data from a 5.405 GHz (C-band) dual-polarized C-band Synthetic Aperture Radar (SAR) instrument with a spatial resolution of 10 m. The GRD data obtained were speckle filtered, radiometrically calibrated, geocoded and data exported by the radar module of SNAP software [44]. We used ArcMap’s resampling tool to resample the preprocessed Sentinel-1A data to 30 m following the NEAREST method. In this way, we obtained two resolutions (10 m and 30 m) of data as input variables in the SOC prediction. Sentinel-2 carries a multispectral sensor instrument (MSI) for terrestrial detection that provides vegetation, soil and water cover imagery from two satellites, 2A and 2B; depending on the time of field sampling, we selected the Sentinel-2A_MSI L1C product data. Level 1-C Sentinel-2 is an orthorectified and geometrically corrected atmospheric surface reflectance product that has not been processed with atmospheric correction. The Level 1-C Sentinel-2 data were therefore converted to surface reflectance data using the ESA Sen2Cor plug-in in SNAP software with a bottom-of-atmosphere (BOA) correction [45]. In this study, 10 bands from Sentinel-2A were extracted to include in the band calculation and modeling, as shown in Table 2. We used the resampling tool with the NEAREST method in ArcMap software to resample the red-edge band, converting the spatial resolution from 20 m to 10 m.

#### 2.3.3. Index Variables for Remote Sensing Data

Constructing a spectral index variable was effective in reducing spectral reflectance errors when building SOC prediction models; therefore, constructing a spectral index to be included as an input variable in an SOC prediction model is considered a fast and effective method. In this study, we selected several vegetation indices, moisture indices and soil brightness index variables to which SOC is sensitive. Among the vegetation indices selected were the normalized difference vegetation index (NDVI) [46,47], enhanced vegetation index (EVI) [48], difference vegetation index (DVI) [49], ratio vegetation index (RVI) [50], transformation vegetation index (TVI) [51], soil-adjusted vegetation index (SAVI) [52], soil-adjusted total vegetation index (SATVI) [53] and normalized burn ratio 2 (NBR2) [19]. The moisture indices include land surface water index (LSWI) [54] and moisture stress index (MSI) [20]. The soil brightness indices include brightness index (BI) [55], brightness index 2 (BI2) [55], redness index (RI) [56] and color index (CI) [56]. These indices were calculated as shown in Table 3. In addition, to compare the spectral differences between Sentinel-2A data and Landsat 8 data, we proposed involving the red-edge indices derived from the red-edge bands of Sentinel-2A in the construction of the SOC prediction model. The red-edge indices derived from the Sentinel-2A data were calculated as shown in Table 4. We referred to these four bands as B5, B6, B7 and B8A, where B5, B6 and B7 replaced B4 and B8A replaced B8; in this way, various combinations of the new red-edge bands were calculated to generate potential spectral indices as input variables for SOC prediction.

#### 2.3.4. Climate Variables

The mean annual temperature (MAT) and mean annual precipitation (MAP) data for the climate variables were obtained from the 0.1 °C and 0.1 mm datasets from the Resource and Environment Data Centre of the Chinese Academy of Sciences (http://www.resdc.cn/, accessed on 14 January 2021). These datasets are based on daily observations from more than 2400 meteorological stations across the country, and national MAT and MAP data (1 km spatial resolution) are generated using the ANUSPLIN interpolation technique. We used the resampling tool in ArcMap software to resample the MAT and MAP data to resolutions of 10 m and 30 m, respectively, following the NEAREST method; this provided a good database with which to determine the accuracy of the climate variables in this study [57].

### 2.4. Modeling Techniques

#### 2.4.1. Random Forest

The machine learning random forest (RF) algorithm is an integrated decision tree-based algorithm that uses booststrap resampling to perform regressions [58]. Booststrap sampling methods reduce the sensitivity of RF to overfitting and thus control the risk of overfitting [59]. RF has become an efficient model widely used in the prediction of soil properties or soil types [32,60]. We implemented the RF algorithm using the Sklearn machine learning library in a Python 3.7 environment. A grid search strategy and a 10-fold cross-validation technique were used to adjust the number and maximum depth of the model regression trees. In this way, the optimal parameters were selected for the prediction of the SOC. RF is a parallel algorithm and its tree model grows in a parallel way. In the RF framework, variable importance is primarily affected by two main parameters: the size of the input variables subset and the number of trees in the forest. Currently, the performance of RF is mostly evaluated by the out-of-bag (OOB) error. Variable importance of $X^{i}$ is then equal to:

$$VI\left(X^{i}\right)=\frac{1}{ntree}\underset{t}{\sum }\left(err{\tilde{OOB}}_{t}{}^{i}-errOOB_{t}\right)$$

where the sum is over all trees t of the RF; ntree denotes the number of trees of the RF; $OOB_{t}$ represents the associated section not included in the bootstrap processes used to construct t; $errOOB_{t}$ is the error of a specific tree t on the associated $OOB_{t}$ sample; and $err{\tilde{OOB}}_{t}{}^{i}$ represents a perturbed sample affected by the permuted values of $X^{i}$.

#### 2.4.2. Gradient Boosted Decision Tree

GBDT are boosting-based tree models that accomplish the regression task through iterative operations [61]. GBDT trains each decision tree in sequence, with each iteration feeding the residuals from the previous decision tree fit into the next decision tree for fitting. In this way, GBDT is highly adaptable and gives good results in soil property inversion. We implemented the algorithm using the Sklearn machine learning library in a Python 3.7 environment. The grid search strategy adjusted the maximum depth of the decision tree, the minimum number of samples required for internal node redivision, the learning rate and the number of regression tree parameters to control the risk of model overfitting. Next, the 10-fold cross-validation technique was used to obtain the optimal GBDT model. In the GBDT algorithm, the global importance of a variable is measured by the average of the importance of feature j in a single tree.

$${\hat{j}}_{j}^{2}=\frac{1}{M}\overset{M}{\underset{m=1}{\sum }}{\hat{j}}_{j}^{2}\left(T_{m}\right)$$

where M is the number of trees. The importance of variable j in a single tree is calculated as follows:

$${\hat{j}}_{j}^{2}\left(T\right)=\overset{L-1}{\underset{t=1}{\sum }}{\hat{I}}_{t}^{2}1\left(v_{t}=j\right)$$

where the L is the number of leaf nodes of the tree, L−1 is the number of non-leaf nodes of the number, $v_{t}$ is the feature associated with node t, and ${\hat{I}}_{t}^{2}$ is the reduction in squared loss after node t splits. When this value is larger, it means that this node has a greater ability to reduce the loss and a greater predictive power.

#### 2.4.3. Extreme Gradient Boosting

Extreme gradient boosting (XGBoost) is a gradient boosting algorithm that has been widely used in regression tasks [62]. XGBoost makes model learning more efficient through parallel computation and uses an additive decision tree training strategy to convert from multiple weak learners to strong learners. With this strategy, XGBoost can handle both classification tasks and regression tasks. In contrast to previous decision tree algorithms, XGBoost is based on a second-order Taylor formula expansion, incorporates a regularization module, and makes predictions through a number of additive functions, effectively controlling the overfitting phenomenon. We implemented the XGBoost algorithm in the Python 3.7 environment using the Sklearn machine learning library. Important hyperparameters such as the number of trees, maximum tree depth, maximum number of nodes or leaves in the decision tree and learning rate are adjusted by means of 10-fold cross-validation and grid search to find the optimal model parameters. In the XGBoost algorithm, the contribution of the corresponding variable through each tree model is obtained using gain, which is calculated as follows:

$$gain=\frac{1}{2}\left[\frac{G_{L}^{2}}{H_{L}+\lambda }+\frac{G_{R}^{2}}{H_{R}+\lambda }-\frac{{\left(G_{L}+G_{R}\right)}^{2}}{H_{L}+H_{R}+\lambda }\right]-\gamma$$

where the $G_{L}$ and $H_{L}$ are associated with the left leaf of the tree model. $\frac{G_{L}^{2}}{H_{L}+\lambda }$ denotes left subtree scores. $G_{R}$ and $H_{R}$ are associated with the right leaf of the tree model. $\frac{G_{R}^{2}}{H_{R}+\lambda }$ denotes right subtree scores. $\frac{{\left(G_{L}+G_{R}\right)}^{2}}{H_{L}+H_{R}+\lambda }$ denotes the score of the node when not split. γ and λ are regularization parameters that prevent overfitting by controlling the simplicity of the tree structure. A higher gain value means that the variable is more important for the prediction of the model.

### 2.5. Modeling Strategy and Validation Metrics

To evaluate and compare the ability of Landsat 8 and Sentinel-2A data to predict SOC, we developed the Landsat 8 and Sentinel-2A common variable models (Model A and Model B) and Sentinel-2A full variable model (Model C). Vegetation, soil moisture, topographic features, precipitation and temperature are the main factors influencing the spatial distribution of SOC [16,63]. The Ebinur Lake wetland is located in an arid and semiarid region with an uneven precipitation distribution and significant topographic variation, so it is necessary to explore the influence of environmental variables on SOC prediction. Sentinel-1A has the advantage of all-weather detection to capture vegetation and soil data, and as a variable added to the SOC prediction model, it can yield good prediction results. To fully consider the effects of different variables on SOC estimation and mapping, different combinations of environmental variables were used to improve prediction accuracy. Therefore, four sub-models were developed under each of the three models, as shown in Table 5. It is worth noting that the difference between Sentinel-2A Model B and Model C was that the remote sensing data variables in Model B did not include the four red-edge bands. This was conducted so that the data corresponded to the six Landsat 8 bands. All 10 band variables were included in Model C. The index variables in Model B included the 14 variables listed in Table 3, while the index variables in Model C included not only the data in Table 3 but also the red-edge index variables in Table 4. The model dataset was divided into a training dataset (70%) and a validation dataset (30%) (test_size = 0.3 in Python 3.7 was used to divide the dataset).

In this study, different forecasting models were evaluated using the following three evaluation metrics: coefficient of determination of use ($R^{2}$), root mean square error (RMSE) and relative prediction error in quartiles (RPIQ). The larger the $R^{2}$ and RPIQ and the smaller the RMSE, the better the prediction accuracy of the model.

(1) $${\;R}^{2}=1-\frac{\sum_{i=1}^{n}{\left(\hat{p_{i}}-p_{i}\right)}^{2}}{\sum_{i=1}^{n}{\left(p_{i}-\bar{p}\right)}^{2}}$$

(2) $$\mathrm{RMSE}=\sqrt{\frac{1}{n}\overset{n}{\underset{i=1}{\sum }}{\left(\hat{p_{i}}-p_{i}\right)}^{2}}$$

(3) $$\mathrm{RPIQ}=\frac{IQ}{{\mathrm{RMSE}}_{P}}$$

where $p_{i}$ is the measured value, $\hat{p_{i}}$ is the predicted value, $\bar{p}$ is the average of the measured values, and n denotes the number of samples. IQ is the interquartile spacing of the measured values in the validation dataset (IQ=Q3−Q1) where Q3 denotes the third quartile and Q1 denotes the first quartile, and ${\mathrm{RMSE}}_{P}$ is the RMSE of the validation dataset.

## 3. Results

### 3.1. Descriptive Statistics

The statistical values of the training and validation datasets for SOC prediction are shown in Table 6. The range of the entire SOC dataset was 1.487–25.015, with a mean of 7.723, median of 7.715 and standard deviation (SD) of 4.380. The final statistics indicated that the overall datasets and the training and validation datasets were similarly distributed. Therefore, the samples in the training and validation datasets were representative of the entire SOC sample and could be used to build and validate an accurate model.

### 3.2. Analysis of the Importance of Variables

**Figure 2:**
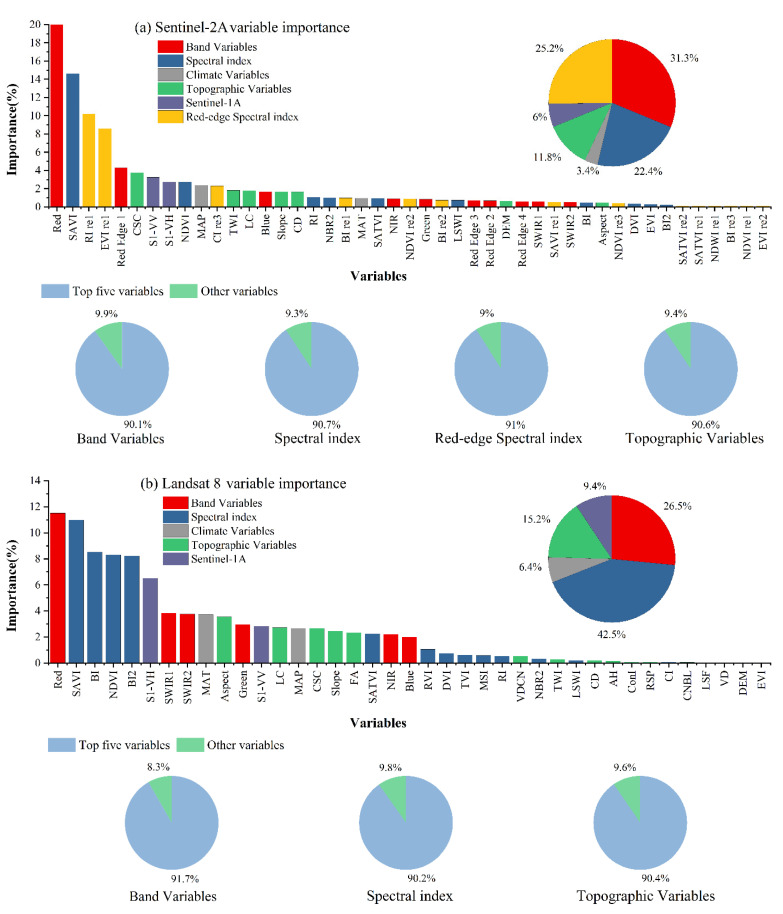
(**a**) Importance of variables in the Landsat 8 model; (**b**) importance of variables in the Sentinel-2A model.

We obtained the importance ranking of each variable in the SOC prediction using the importance calculation of the RF algorithm. The RF variable preference method is widely used and has better generalization capabilities. In Python 3.7, we calculated the importance values for each feature by way of Sklearn using the feature importance function. To increase comparability between variables, we normalized the importance of the variables to 100% and analyzed the extent to which the modeled variables influenced the SOC predictions for this study area (Figure 2a,b). We counted the share of each type of variable among all variables according to their importance and separately in each category itself. In the Landsat 8 model, the top five variables in band variables, spectral index variables and topographic variables accounted for 91.7%, 90.2% and 90.4%, respectively. In the Sentinel-2A model, the top five variables in band variables, spectral index variables, red-edge spectral index variables and topographic variables reached 90.1%, 90.7%, 91% and 90.6%, respectively. We ultimately found that the top five accounted for over 90% of the variables in each category. The top five variables can represent this category of variables well for modeling and prediction. The Landsat 8 model ranked Red, SWIR1, SWIR2, Green and NIR in the top five in terms of importance of band variables by variable importance. SAVI, BI, NDVI, BI2 and SATVI ranked in the top five for the importance of the spectral index variables. Aspect, LC, CSC, Slope and FA ranked in the top five for the importance of the topographic variables. The Sentinel-2A model has Red, RE 1, Blue, NIR and Green in the top five for the importance of the band variables. SAVI, NDVI, RI, NBR2 and SATVI ranked in the top five for the importance of the spectral index variables. RI re1, EVI re1, CI re3, BI re1 and NDVI re2 ranked in the top five in importance for the red-edge spectral index variables. CSC, TWI, LC, Slope and CD ranked in the top five in importance for the topography variables. We found the highest proportion of spectral index variables (42.5%) in the Landsat 8 model (Figure 2a), with SAVI being the most important, followed by BI, NDVI and BI2. The band index variables accounted for 26.5% of the importance of the variables, with Red being the most important, followed by SWIR1, SWIR2,Green and NIR. The topographic variables accounted for 15.2% of the importance of the variables, with the aspect being the most important topographic variable, followed by LC, CSC, Slope and FA. In the full variable Sentinel-2A model (Figure 2b), the band variables accounted for the greatest proportion (31.3%) of the importance, with the Red band being most important, followed by RE 1. The spectral index variables accounted for 22.4% of the total importance, with SAVI having the highest importance, followed by NDVI. The red-edge index accounted for 25.2% of the importance, with RI re1 having the highest importance, followed by EVI re1.Topographic variables accounted for 11.8% of the importance, with CSC being the most important of the topographic variables, followed by TWI, LC, Slope and CD topographic variables.

In the Landsat 8 model, the sum of the importance of the band and index variables for remotely sensed data was 69%. In the full variable Sentinel-2A model, the sum of the importance of the band variables and index variables was 78.9%. Overall, remotely sensed data and derived variables were significantly more important than other variables in the SOC predictions, and the importance of data derived from Sentinel-2A was greater than that of data derived from Landsat 8. 

### 3.3. Comparison of the Performances of Different Models

To compare the effects of different combinations of variables on the SOC prediction results, the predictive performances of the three machine learning algorithms (RF, GBDT and XGBoost) were assessed. In the SOC prediction model, valuable variables will improve the prediction accuracy of the model, but a large number of variables with low importance will reduce the prediction accuracy of the model. We therefore used all variables for modeling and prediction, and we selected the top five variables in each category for modeling and prediction based on the variable importance results. This is because the top five variables in each category accounted for more than 90% of the explanatory power in the variable importance results and can represent this category of variables well. Based on the modeling of these two sets of experiments, we obtained the final results, as shown in Table 7. The results, where all variables were involved in the modeling, showed that Model A-IV with the XGBoost algorithm had the best predictive performance ($R^{2}$ = 0.701, RMSE = 2.291, RPIQ = 2.007) among the models built with Landsat 8 (Model A). The $R^{2}$ of the XGBoost models was improved by 2% and 1.2% compared with those of the RF and GBDT algorithms, respectively. In the Sentinel-2A common variable model (Model B), Model B-IV created with the XGBoost algorithm had the best prediction performance ($R^{2}$ = 0.735, RMSE = 2.037, RPIQ = 2.337), with the XGBoost algorithm improving the model $R^{2}$ by 3.4% and 2.7% compared with those of the RF and GBDT models, respectively. In the Sentinel-2A full variable model (Model C) with the addition of the red-edge band and red-edge index, Model C-IV combined with the XGBoost algorithm had the best predictive performance ($R^{2}$ = 0.771, RMSE = 1.899, RPIQ = 2.506), with the XGBoost algorithm improving the model $R^{2}$ by 6.6% and 2% compared with those of the RF and GBDT models, respectively. The modeling results using the top five variables in each category showed that of the models built with Landsat 8 (Model A), Model A-IV built with the XGBoost algorithm had the best predictive performance ($R^{2}$ = 0.759, RMSE = 2.033, RPIQ = 2.376). The $R^{2}$ of the XGBoost models was improved by 5% and 3.6% compared with those of the RF and GBDT algorithms, respectively. In the Sentinel-2A common variable model (Model B), Model B-IV created with the XGBoost algorithm had the best prediction performance ($R^{2}$ = 0.788, RMSE = 1.921, RPIQ = 2.477), with the XGBoost algorithm improving the model $R^{2}$ by 5.2% and 3.5% compared with those of the RF and GBDT models, respectively. In the Sentinel-2A full variable model (Model C) with the addition of the red-edge band and red-edge index, Model C-IV combined with the XGBoost algorithm had the best predictive performance ($R^{2}$ = 0.804, RMSE = 1.771, RPIQ = 2.687), with the XGBoost algorithm improving the model $R^{2}$ by 6% and 3.2% compared with those of the RF and GBDT models, respectively. In addition, the prediction accuracy of the different models increased with increasing environmental variables under the XGBoost algorithm. The optimal $R^{2}$ for the common variable Landsat 8 model was 0.709, 0.723 and 0.759 under the RF, GBDT and XGBoost algorithms, respectively. The optimal $R^{2}$ for the common variable Sentinel-2A model was 0.736, 0.753 and 0.788, respectively. The optimal $R^{2}$ for the full variable Sentinel-2A model was 0.744, 0.772 and 0.804, respectively.

Overall, better predictions were achieved using the first five variables after variable filtering to complete the modeling. Compared to full variable modeling, the RF best $R^{2}$ improved by 3.9%, the GBDT best $R^{2}$ improved by 2.1% and the XGBoost best $R^{2}$ improved by 3.3%. Among the three machine learning algorithms, the XGBoost algorithm provided better predictions than the GBDT algorithm and the RF algorithm, and the GBDT algorithm performed better than the RF algorithm. In terms of the combination of variable factors, the combination of remote sensing data band variables, index variables of remote sensing data, terrain variables, climate variables and Sentinel-1A could provide better prediction results. The red-edge variables involved in the modeling had better prediction accuracy than the other models. In the top five variable models, the contribution of the red-edge variables resulted in better predictions for Model C-III than Model B-III. Model C-III improved $R^{2}$ by 2.8%, 1.4% and 3.2%, respectively, over Model B-III under the three machine learning algorithms.

### 3.4. SOC Spatial Predictions from Landsat 8 and Sentinel-2A

**Figure 3:**
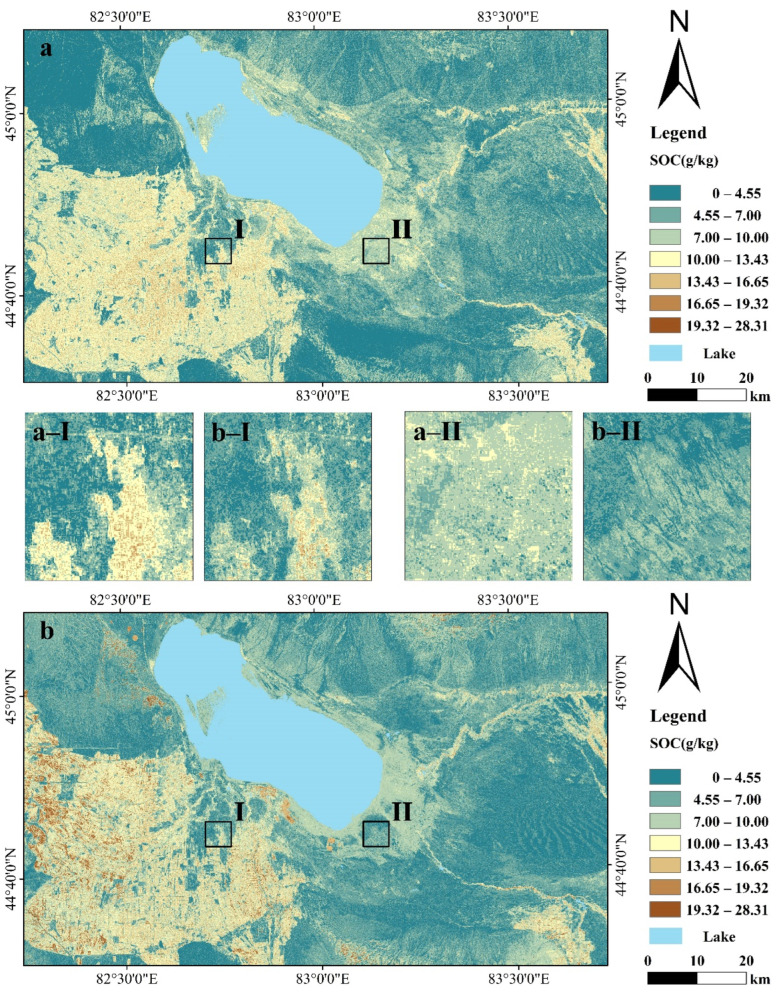
(**a**) Spatial distribution of SOC content predicted using the Landsat 8 model; (**b**) spatial distribution of SOC content predicted using the full variable Sentinel-2A model; (**a**-**I**), (**a**-**II**), (**b**-**I**) and (**b-II**) show specific details in an area for comparison.

We used the XGBoost algorithm with the best prediction results to generate a model of the spatial distribution of SOC in the study area (Figure 3a,b). By comparing Figure 3a,b, we found that the overall trends of the spatial distribution of SOC on the maps created with Landsat 8 and Sentinel-2A data were similar, showing that the areas with high SOC contents were mainly concentrated in the oasis and that the oasis SOC content was higher than that around Ebinur Lake and in the mountainous area. The model based on Sentinel-2A data predicted SOC contents of approximately 10.00–28.31 g/kg in the interior of the oasis, while the SOC contents in the interior of the oasis were generally predicted to be lower by the model based on Landsat 8 data, with SOC contents ranging from 10.00 g/kg to 19.32 g/kg. Comparisons of Figure 3a-I,a-II,b-I,b-II show that the Sentinel-2A mapping results were much clearer than the Landsat 8 mapping results, making it easier for us to observe the spatial distribution of the SOC content. Overall, the models based on Sentinel-2A data provided a better prediction of the spatial distribution of SOC content, showing greater differences in the spatial distribution of SOC in both detail and overall.

## 4. Discussion

### 4.1. Comparison of Models Based on Landsat 8 and Sentinel-2A Data

With the development of remote sensing technology, various multispectral sensors are able to obtain useful geographic information based on the reflected spectral information of target objects on the land surface. Therefore, multisource satellite remote sensing data provide new perspectives and possibilities for SOC spatial mapping. In this study, Landsat 8 and Sentinel-2A common variables and Sentinel-2A full variables were used to build the models, respectively. This comparison method of common variables and full variables can effectively compare the differences between different remote sensing data. When the Landsat 8 and Sentinel-2A models created with the same variables were applied for SOC prediction, the Sentinel-2A model performed better, which may be because the Sentinel-2A data (10 m) have a higher spatial resolution than the Landsat 8 data (30 m). More mixed image elements may be included at a spatial resolution of 30 m [64], which reduces the SOC prediction accuracy. Fabio, C. obtained similar results when estimating topsoil properties in agricultural fields [19]. The predictive performances of the models based on Landsat 8 and Sentinel-2A data were similar under the three ensemble learning algorithms. The majority of the models with $R^{2}\;$ > 0.7 were in the sub-model IV. This phenomenon is most evident in the XGBoost algorithm, which has good prediction results for all models ($R^{2}\;$ > 0.7) except Model A-I in the Landsat 8 model. The full variable Sentinel-2A models predicted better performance than the common variable Sentinel-2A models at the same resolution. This difference may be due to the inclusion of the red-edge band and the red-edge index in the full variable Sentinel-2A models. The significance analyses we conducted supported this result, with the red-edge index accounting for a significant share of the variables predicting SOC; indeed, there has been strong support for the use of red-edge bands and derived red-edge indices for modeling soil properties [65,66]. This study speculates that the red-edge index exploits the potential of the Sentinel-2A band combination to provide more effective spectral information for SOC prediction models. Overall, a prediction model with high spatial resolution and finer spectral information is the optimal choice for SOC mapping.

### 4.2. Analysis of Environmental Variables

Indicators of the importance of environmental variables are shown in Figure 2. We found that remotely sensed data and derived index variables had the highest proportions in the Landsat 8 and Sentinel-2A models (68.9% and 74%). This is mainly because vegetation and soil characteristics are the dominant factors in determining SOC content under similar climatic conditions [67]. Therefore, vegetation indices derived from remotely sensed data, soil moisture and brightness indices can represent vegetation biomass and soil characteristics [36,68]. Wang, K. found NDVI, BI, BI2 and SATVI to be the most important variables for predicting farmland SOC in autumn [69], which is consistent with the results of this paper. Due to the sensitivity of SAVI to soil properties [70], it is considered the most important variable in agricultural soil organic carbon prediction. Topographic variables accounted for a large proportion of the variables in this study (Figure 2). Some studies have shown that the spatial distribution of SOC content is significantly influenced by topographic factors [71], showing a specific distribution trend [72]; these findings are consistent with those of this paper. Among the topographic variables, LC links the erosion and spatial distribution of surface material, reflecting the soil profile and geological structure [73]. In studies of SOC contents, the CSC can be applied to distinguish areas of lower elevation and less pronounced topographic variation, which are not conducive to humus accumulation. The introduction of the CSC has improved the accuracy of soil identification [74]. TWI is an effective topographic variable affecting the spatial distribution of SOC contents. It has been shown that on steep slopes and in uphill areas, the accumulation of SOC increases with increasing TWI values [75]. In this study, the FA, Slope and aspect likewise provide valuable information for SOC prediction, each with a certain degree of contribution. These DEM-derived variables were used in the studies of Dharumarajan, S. and Kabindra, A. as important topographic factors for predicting the spatial distribution of SOC contents [76,77]. In the present study, Sentinel-1A was used as a variable to obtain better SOC prediction performance than a single optical image model when combined with optical images. This finding is consistent with those of previous research [36]. Vegetation productivity is considered to be an important factor in the spatial distribution of soil organic carbon because it determines the amount of organic carbon input, so capturing information on vegetation and soils is essential for SOC prediction [78]. SAR backscatters better reflect soil attributes, and SAR data can effectively identify the relationship between vegetation and soil organic carbon [79]. In addition, the quality of SAR data is independent of weather and daylight and is an important dataset for explaining spatial variability in soil properties [80]. Sentinel-1A, as freely available SAR data, offers better opportunities for soil property estimation. Most of the current studies use a single optical image combined with SAR data, thus obtaining better results [81,82,83]. Combinations including more sensor data should be considered in the future, but the uniformity of spatial resolution between different sensors will be a challenge for image processing by image calculation and mapping. In this study, a high SOC content was found in the interior of the oasis, which we speculate is due to frequent agricultural activities and land use.

### 4.3. Uncertainty Analysis

In this study, we chose three machine learning algorithms (RF, GBDT and XGBoost) to predict the SOC content in the study area. Although RF, GBDT and XGBoost are all ensemble learning algorithms, there are differences in their interpretation of multisource remote sensing data. Among the three machine learning algorithms, RF is only a basic parallel algorithm. GBDT uses only first-order derivative information in the optimization process. XGBoost uses a second-order Taylor expansion, using both first- and second-order derivatives. Therefore, among the three ensemble learning algorithms, XGBoost had the best prediction performance ($R^{2}$ = 0.804, RMSE = 1.771, RPIQ = 2.687), which is consistent with studies by Liang and Nguyen [2,38]. The XGBoost algorithm has demonstrated excellent prediction results in recent studies [84,85]. Landsat 8 and Sentinel-2A data are predicted with higher accuracy by the XGBoost algorithm, which is supported by the findings of recent studies [86,87]. For machine learning, more variables are not better, and even though XGBoost achieved the best results, the prediction was only slightly improved in the model with the addition of environmental variables. If environmental variables are costly to obtain, relatively good and similar results can be obtained by using SOC estimation performed with spectral information. This consideration causes a slight loss of model accuracy while reducing the high cost of prediction, as Sentinel-2A data can provide finer spatial resolution and effective spectral information [20]. Furthermore, the advantage of ensemble learning is that the potential relationships between data and variables can be fully explored through an ensemble of multiple weak learners. There will be differences in the selection of variables in the models constructed by machine learning algorithms. Because different machine learning algorithms calculate the importance of variables in different ways, these algorithms also differ in the way they decide on decision tree splits. The uncertainty that this bias creates in the predictive performance of machine learning models is inevitable. We can control that the input variables and samples are the same during the input of different algorithms to reduce this uncertainty as much as possible. In future research, the applicability of ensemble learning algorithms should be considered and the XGBoost algorithm should be applied to different study areas as a way to validate the applicability of the XGBoost algorithm.

## 5. Conclusions

The common variable models of Landsat 8 with Sentinel-2A and the full variable model of Sentinel-2A were constructed to assess the superiority of Sentinel-2A data in soil organic carbon (SOC) prediction models. Sentinel-2A data provided more effective red-edge information, such as constructed red-edge indices to joint SOC and spectral remote sensing. Compared with the models constructed with Landsat 8 data, the models constructed with Sentinel-2 data demonstrated superior estimation accuracy and more legible mapped details. This highlighted that SOC spatial estimation relies on finer spatial and spectral resolutions. In addition, the majority of the IV sub-models predicted better performance in all models, with $R^{2}\;$ > 0.7 for all IV sub-models in the extreme gradient boosting (XGBoost) algorithm. The XGBoost algorithm was a powerful machine learning algorithm ($R^{2}$ = 0.637~0.804) and outperformed the random forest (RF) models ($R^{2}$ = 0.606~0.744) and gradient boosted decision tree (GBDT) models ($R^{2}$ = 0.641~0.772). We noticed that the spatial distribution of the SOC content in the Ebinur Lake wetland varied significantly. The oasis was predicted to have a higher SOC content, while the foothills and lakeshore had lower SOC contents. In conclusion, the combination of a Sentinel-2-driven multisource remote sensing modeling strategy and XGBoost enables reliable assessment of SOC, which provides strong support for local governments to formulate appropriate carbon sequestration policies to prevent land degradation and ultimately achieve sustainable development goals.

## Figures and Tables

**Table 1:** Landsat 8 and Sentinel-2A data band information.

Satellite Sensor Name	Band Name	Spectral Position (nm)	Central Wavelength (nm)	Original Resolution (m)
Sentinel-2A/MSI	B2-Blue	458–523	490	10
	B3-Green	543–578	560	10
	B4-Red	650–680	665	10
	B5-Red Edge 1	698–713	705	20
	B6-Red Edge 2	733–748	740	20
	B7-Red Edge 3	773–793	783	20
	B8-NIR	785–900	842	10
	B8A-Red Edge 4	855–875	865	20
	B11-SWIR1	1565–1655	1610	20
	B12-SWIR2	2100–2280	2190	20
Landsat 8/OLI	B2-Blue	450–515	483	30
	B3-Green	525–600	560	30
	B4-Red	630–680	660	30
	B5-NIR	845–885	865	30
	B6-SWIR1	1560–1660	1650	30
	B7-SWIR2	2100–2300	2220	30

**Table 2:** Sentinel-1A data information.

Date	Sensor Mode	Polarization	Direction
26 July 2017	IW	VV	Ascending
26 July 2017	IW	VH	Ascending

**Table 3:** Spectral index information for Landsat 8 and Sentinel-2A data.

Index	Formula	Sentinel-2A MSI Equation	Landsat 8 OIL Equation
NDVI	$\frac{\mathrm{NIR}\;-\;R}{\mathrm{NIR}\;+\;R}$	$\frac{B8\;-\;B4}{B8\;+\;B4}$	$\frac{B5\;-\;B4}{B5\;+\;B4}$
VI	$2.5\;\times \;\frac{\mathrm{NIR}\;-\;R}{\mathrm{NIR}\;+\;6\;\times \;R\;-\;7.5\;\times \;B\;+\;1}$	$2.5\;\times \;\frac{B8\;-\;B4}{B8\;+\;6\;\times \;B4\;-\;7.5\;\times \;B2\;+\;1}$	$2.5\;\times \;\frac{B5\;-\;B4}{B5\;+\;6\;\times \;B4\;-\;7.5\;\times \;B2\;+\;1}$
VI	NIR − R	B8 − B4	B5 − B4
VI	$\frac{\mathrm{NIR}}{R}$	$\frac{B8}{B4}$	$\frac{B5}{B4}$
TVI	$\sqrt{\frac{\mathrm{NIR}\;-\;R}{\mathrm{NIR}\;+\;R}\;+\;0.5}\;\times \;100$	$\sqrt{\frac{B8\;-\;B4}{B8\;+\;B4}\;+\;0.5}\;\times \;100$	$\sqrt{\frac{B5\;-\;B4}{B5\;+\;B4}\;+\;0.5}\;\times \;100$
SAVI	$\frac{\left(\mathrm{NIR}\;-\;R\right)\;\times \;1.5}{\mathrm{NIR}\;+\;R\;+\;0.5}$	$\frac{\left(B8\;-\;B4\right)\;\times \;1.5}{B8\;+\;B4\;+\;0.5}$	$\frac{\left(B5\;-\;B4\right)\;\times \;1.5}{B5\;+\;B4\;+\;0.5}$
SATVI	$\frac{\mathrm{SWIR}1\;-\;R}{\mathrm{SWIR}1\;+\;R\;+\;1}\;\times \;2\;-\;\frac{\mathrm{SWIR}2}{2}$	$\frac{B11\;-\;B4}{B11\;+\;B4\;+\;1}\;\times \;2\;-\;\frac{B12}{2}$	$\frac{B6\;-\;B4}{B6\;+\;B4\;+\;1}\;\times \;2\;-\;\frac{B7}{2}$
NBR2	$\frac{\mathrm{SWIR}1\;-\;\mathrm{SWIR}2}{\mathrm{SWIR}1\;+\;\mathrm{SWIR}2}$	$\frac{B11\;-\;B12}{B11\;+\;B12}$	$\frac{B6\;-\;B7}{B6\;+\;B7}$
BI	$\frac{\sqrt{\left(R\;\times \;R\right)\;+\;\left(G\;\times \;G\right)}}{2}$	$\frac{\sqrt{\left(B4\;\times \;B4\right)\;+\;\left(B3\;\times \;B3\right)}}{2}$	$\frac{\sqrt{\left(B4\;\times \;B4\right)\;+\;\left(B3\;\times \;B3\right)}}{2}$
BI2	$\frac{\sqrt{\left(R\;\times \;R\right)\;+\;\left(G\;\times \;G\right)\;+\;(\mathrm{NIR}\;\times \;\mathrm{NIR})}}{2}$	$\frac{\sqrt{\left(B4\;\times \;B4\right)\;+\;\left(B3\;\times \;B3\right)\;+\;(B8\;\times \;B8)}}{2}$	$\frac{\sqrt{\left(B4\;\times \;B4\right)\;+\;\left(B3\;\times \;B3\right)\;+\;(B5\;\times \;B5)}}{2}$
RI	$\frac{R\;\times \;R}{G\;\times \;G\;\times \;G}$	$\frac{B4\;\times \;B4}{B3\;\times \;B3\;\times \;B3}$	$\frac{B4\;\times \;B4}{B3\;\times \;B3\;\times \;B3}$
CI	$\frac{R\;-\;G}{R\;+\;G}$	$\frac{B4\;-\;B3}{B4\;+\;B3}$	$\frac{B4\;-\;B3}{B4\;+\;B3}$
LSWI	$\frac{\mathrm{NIR}\;-\;\mathrm{SWIR}1}{\mathrm{NIR}\;+\;\mathrm{SWIR}1}$	$\frac{B8\;-\;B11}{B8\;+\;B11}$	$\frac{B5\;-\;B6}{B5\;+\;B6}$
MSI	$\frac{\mathrm{SWIR}1}{\mathrm{NIR}}$	$\frac{B11}{B8}$	$\frac{B6}{B5}$

**Table 4:** The newly proposed red-edge spectral indices.

Index	Formula	Sentinel-2 MSI Equation
NDVI re1	$\frac{\mathrm{RE}\;4\;-\;\mathrm{RE}\;1}{\mathrm{RE}\;4\;+\;\mathrm{RE}\;1}$	$\frac{B8A\;-\;B5}{B8A\;+\;B5}$
NDVI re2	$\frac{\mathrm{RE}\;4\;-\;\mathrm{RE}\;2}{\mathrm{RE}\;4\;+\;\mathrm{RE}\;2}$	$\frac{B8A\;-\;B6}{B8A\;+\;B6}$
NDVI re3	$\frac{\mathrm{RE}\;4\;-\;\mathrm{RE}\;3}{\mathrm{RE}\;4\;+\;\mathrm{RE}\;3}$	$\frac{B8A\;-\;B7}{B8A\;+\;B7}$
EVI re1	$2.5\;\times \;\frac{\mathrm{RE}\;4\;-\;\mathrm{RE}\;1}{\mathrm{RE}\;4\;+\;6\;\times \;\mathrm{RE}\;1\;-\;7.5\;\times \;B\;+\;1}$	$2.5\;\times \;\frac{B8A\;-\;B5}{B8A\;+\;6\;\times \;B5\;-\;7.5\;\times \;B2\;+\;1}$
EVI re2	$2.5\;\times \;\frac{\mathrm{RE}\;4\;-\;\mathrm{RE}\;2}{\mathrm{RE}\;4\;+\;6\;\times \;\mathrm{RE}\;2\;-\;7.5\;\times \;B\;+\;1}$	$2.5\;\times \;\frac{B8A\;-\;B6}{B8A\;+\;6\;\times \;B6\;-\;7.5\;\times \;B2\;+\;1}$
EVI re3	$2.5\;\times \;\frac{\mathrm{RE}\;4\;-\;\mathrm{RE}\;3}{\mathrm{RE}\;4\;+\;6\;\times \;\mathrm{RE}\;3\;-\;7.5\;\times \;B\;+\;1}$	$2.5\text{×}\frac{B8A\;-\;B7}{B8A\;+\;6\;\times \;B7\;-\;7.5\;\times \;B2\;+\;1}$
DVI re1	RE 4 − RE 1	B8A − B5
DVI re2	RE 4 − RE 2	B8A − B6
DVI re3	RE 4 − RE 3	B8A − B7
RVI re1	$\frac{\mathrm{RE}\;4}{\mathrm{RE}\;1}$	$\frac{B8A}{B5}$
RVI re2	$\frac{\mathrm{RE}\;4}{\mathrm{RE}\;2}$	$\frac{B8A}{B6}$
RVI re3	$\frac{\mathrm{RE}\;4}{\mathrm{RE}\;3}$	$\frac{B8A}{B7}$
TVI re1	$\sqrt{\frac{\mathrm{RE}\;4\;-\;\mathrm{RE}\;1}{\mathrm{RE}\;4\;+\;\mathrm{RE}\;1}\;+\;0.5}\;\times \;100$	$\sqrt{\frac{B8A\;-\;B5}{B8A\;+\;B5}\;+\;0.5}\;\times \;100$
TVI re2	$\sqrt{\frac{\mathrm{RE}\;4\;-\;\mathrm{RE}\;2}{\mathrm{RE}\;4\;+\;\mathrm{RE}\;2}\;+\;0.5}\;\times \;100$	$\sqrt{\frac{B8A\;-\;B6}{B8A\;+\;B6}\;+\;0.5}\;\times \;100$
TVI re3	$\sqrt{\frac{\mathrm{RE}\;4\;-\;\mathrm{RE}\;3}{\mathrm{RE}\;4\;+\;\mathrm{RE}\;3}\;+\;0.5}\;\times \;100$	$\sqrt{\frac{B8A\;-\;B7}{B8A\;+\;B7}\;+\;0.5}\;\times \;100$
SAVI re1	$\frac{\left(\mathrm{RE}\;4\;-\;\mathrm{RE}\;1\right)\;\times \;1.5}{\mathrm{RE}\;4\;+\;\mathrm{RE}\;1\;+\;0.5}$	$\frac{\left(B8A\;-\;B5\right)\;\times \;1.5}{B8A\;+\;B5\;+\;0.5}$
SAVI re2	$\frac{\left(\mathrm{RE}\;4\;-\;\mathrm{RE}\;2\right)\;\times \;1.5}{\mathrm{RE}\;4\;+\;\mathrm{RE}\;2\;+\;0.5}$	$\frac{\left(B8A\;-\;B6\right)\;\times \;1.5}{B8A\;+\;B6\;+\;0.5}$
SAVI re3	$\frac{\left(\mathrm{RE}\;4\;-\;\mathrm{RE}\;3\right)\;\times \;1.5}{\mathrm{RE}\;4\;+\;\mathrm{RE}\;3\;+\;0.5}$	$\frac{\left(B8A\;-\;B7\right)\;\times \;1.5}{B8A\;+\;B7\;+\;0.5}$
SATVI re1	$\frac{\mathrm{SWIR}1\;-\;\mathrm{RE}\;1}{\mathrm{SWIR}1\;+\;\mathrm{RE}\;1\;+\;1}\;\times \;2\;-\;\frac{\mathrm{SWIR}2}{2}$	$\frac{B11\;-\;B5}{B11\;+\;B5\;+\;1}\;\times \;2\;-\;\frac{B12}{2}$
SATVI re2	$\frac{\mathrm{SWIR}1\;-\;\mathrm{RE}\;2}{\mathrm{SWIR}1\;+\;\mathrm{RE}\;2\;+\;1}\;\times \;2\;-\;\frac{\mathrm{SWIR}2}{2}$	$\frac{B11\;-\;B6}{B11\;+\;B6\;+\;1}\;\times \;2\;-\;\frac{B12}{2}$
SATVI re3	$\frac{\mathrm{SWIR}1\;-\;\mathrm{RE}\;3}{\mathrm{SWIR}1\;+\;\mathrm{RE}\;3\;+\;1}\;\times \;2\;-\;\frac{\mathrm{SWIR}2}{2}$	$\frac{B11\;-\;B7}{B11\;+\;B7\;+\;1}\;\times \;2\;-\;\frac{B12}{2}$
BI re1	$\frac{\sqrt{\left(\mathrm{RE}\;1\;\times \;\mathrm{RE}\;1\right)\;+\;\left(G\;\times \;G\right)}}{2}$	$\frac{\sqrt{\left(B5\;\times \;B5\right)\;+\;\left(B3\;\times \;B3\right)}}{2}$
BI re2	$\frac{\sqrt{\left(\mathrm{RE}\;2\;\times \;\mathrm{RE}\;2\right)\;+\;\left(G\;\times \;G\right)}}{2}$	$\frac{\sqrt{\left(B6\;\times \;B6\right)\;+\;\left(B3\;\times \;B3\right)}}{2}$
BI re3	$\frac{\sqrt{\left(\mathrm{RE}\;3\;\times \;\mathrm{RE}\;3\right)\;+\;\left(G\;\times \;G\right)}}{2}$	$\frac{\sqrt{\left(B7\;\times \;B7\right)\;+\;\left(B3\;\times \;B3\right)}}{2}$
BI2 re1	$\frac{\sqrt{\left(\mathrm{RE}\;1\;\times \;\mathrm{RE}\;1\right)\;+\;\left(G\;\times \;G\right)\;+\;(\mathrm{RE}\;4\;\times \;\mathrm{RE}\;4)}}{2}$	$\frac{\sqrt{\left(B5\;\times \;B5\right)\;+\;\left(B3\;\times \;B3\right)\;+\;(B8A\;\times \;B8A)}}{2}$
BI2 re2	$\frac{\sqrt{\left(\mathrm{RE}\;2\;\times \;\mathrm{RE}\;2\right)\;+\;\left(G\;\times \;G\right)\;+\;(\mathrm{RE}\;4\;\times \;\mathrm{RE}\;4)}}{2}$	$\frac{\sqrt{\left(B6\;\times \;B6\right)\;+\;\left(B3\;\times \;B3\right)\;+\;(B8A\;\times \;B8A)}}{2}$
BI2 re3	$\frac{\sqrt{\left(\mathrm{RE}\;3\;\times \;\mathrm{RE}\;3\right)\;+\;\left(G\;\times \;G\right)\;+\;(\mathrm{RE}\;4\;\times \;\mathrm{RE}\;4)}}{2}$	$\frac{\sqrt{\left(B7\;\times \;B7\right)\;+\;\left(B3\;\times \;B3\right)\;+\;(B8A\;\times \;B8A)}}{2}$
RI re1	$\frac{\mathrm{RE}\;1\;\times \;\mathrm{RE}\;1}{G\;\times \;G\;\times \;G}$	$\frac{B5\;\times \;B5}{B3\;\times \;B3\;\times \;B3}$
RI re2	$\frac{\mathrm{RE}\;2\;\times \;\mathrm{RE}\;2}{G\;\times \;G\;\times \;G}$	$\frac{B6\;\times \;B6}{B3\;\times \;B3\;\times \;B3}$
RI re3	$\frac{\mathrm{RE}\;3\;\times \;\mathrm{RE}\;3}{G\;\times \;G\;\times \;G}$	$\frac{B7\;\times \;B7}{B3\;\times \;B3\;\times \;B3}$
CI re1	$\frac{\mathrm{RE}\;1\;-\;G}{\mathrm{RE}\;1\;+\;G}$	$\frac{B5\;-\;B3}{B5\;+\;B3}$
CI re2	$\frac{\mathrm{RE}\;2\;-\;G}{\mathrm{RE}\;2\;+\;G}$	$\frac{B6\;-\;B3}{B6\;+\;B3}$
CI re3	$\frac{\mathrm{RE}\;3\;-\;G}{\mathrm{RE}\;3\;+\;G}$	$\frac{B7\;-\;B3}{B7\;+\;B3}$
NDWI re1	$\frac{\mathrm{RE}\;4\;-\;\mathrm{SWIR}1}{\mathrm{RE}\;4\;+\;\mathrm{SWIR}1}$	$\frac{B8A\;-\;B11}{B8A\;+\;B11}$
MSI re1	$\frac{\mathrm{SWIR}1}{\mathrm{RE}\;4}$	$\frac{B11}{B8A}$

**Table 5:** Details of the Landsat 8 and Sentinel-2A modeling strategies.

Model Name	Variable Combinations
Model A-I	Landsat 8(6band)
Model A-II	Landsat 8(6band) + Spectral index
Model A-III	Landsat 8(6band) + Spectral index + Climate variables + Topographic variables
Model A-IV	Landsat 8(6band) + Spectral index + Climate variables + Topographic variables + Sentinel-1A
Model B-I	Sentinel-2A(6band)
Model B-II	Sentinel-2A(6band) + Spectral index
Model B-III	Sentinel-2A(6band) + Spectral index + Climate variables + Topographic variables
Model B-IV	Sentinel-2A(6band) + Spectral index + Climate variables + Topographic variables + Sentinel-1A
Model C-I	Sentinel-2A(10band)
Model C-II	Sentinel-2A(10band) + Spectral index + Red-edge index
Model C-III	Sentinel-2A(10band) + Spectral index + Red-edge index + Climate variables + Topographic variables
Model C-IV	Sentinel-2A(10band) + Spectral index + Red-edge index + Climate variables + Topographic variables + Sentinel-1A

**Table 6:** Descriptive statistics for the entire SOC dataset, the training dataset and the validation dataset.

Dataset	Sample Size	Minimum (g/kg)	Maximum (g/kg)	Median (g/kg)	Mean (g/kg)	Standard Deviation (g/kg)
Whole dataset	95	1.487	25.015	7.715	7.723	4.380
Training dataset	66	1.487	25.015	7.889	7.905	4.582
Validation dataset	29	1.699	17.245	7.036	7.310	3.926

**Table 7:** Predictive performances of the models.

Modeling Technique	Model Name	All Variables			Top 5 Variables		
		$R^{2}$	RMSE (g/kg)	RPIQ	$R^{2}$	RMSE (g/kg)	RPIQ
RF	Model A-I	0.583	2.781	1.711	0.606	2.474	1.924
	Model A-II	0.633	2.692	1.768	0.648	2.343	2.031
	Model A-III	0.627	2.640	1.803	0.661	2.299	2.070
	Model A-IV	0.681	2.447	1.945	0.709	2.141	2.223
	Model B-I	0.615	2.660	1.789	0.624	2.617	1.818
	Model B-II	0.632	2.502	1.902	0.655	2.426	1.962
	Model B-III	0.569	2.537	1.876	0.685	2.179	2.184
	Model B-IV	0.701	2.401	1.982	0.736	2.067	2.303
	Model C-I	0.615	2.596	1.833	0.654	2.252	1.885
	Model C-II	0.693	2.405	1.979	0.694	2.230	2.135
	Model C-III	0.640	2.387	1.994	0.713	2.148	2.216
	Model C-IV	0.705	2.106	2.260	0.744	2.005	2.374
GBDT	Model A-I	0.531	2.630	1.810	0.641	2.393	1.976
	Model A-II	0.689	2.463	1.933	0.695	2.237	2.128
	Model A-III	0.670	2.369	2.009	0.704	2.236	2.129
	Model A-IV	0.671	2.374	2.004	0.723	2.132	2.233
	Model B-I	0.626	2.483	1.917	0.654	2.367	2.010
	Model B-II	0.649	2.364	2.014	0.699	2.244	2.121
	Model B-III	0.681	2.229	2.135	0.713	2.110	2.255
	Model B-IV	0.708	2.132	2.232	0.753	2.057	2.334
	Model C-I	0.659	2.347	2.028	0.682	2.238	2.126
	Model C-II	0.663	2.370	2.008	0.708	2.190	2.174
	Model C-III	0.687	2.267	2.100	0.727	2.084	2.284
	Model C-IV	0.751	2.104	2.262	0.772	1.965	2.423
XGBoost	Model A-I	0.600	2.483	1.917	0.637	2.327	2.045
	Model A-II	0.677	2.394	1.988	0.702	2.155	2.209
	Model A-III	0.693	2.420	1.966	0.726	2.124	2.241
	Model A-IV	0.701	2.291	2.077	0.759	2.003	2.376
	Model B-I	0.685	2.236	2.129	0.701	2.175	2.188
	Model B-II	0.693	2.342	2.033	0.722	2.242	2.223
	Model B-III	0.712	2.111	2.254	0.754	1.987	2.395
	Model B-IV	0.735	2.037	2.337	0.788	1.921	2.477
	Model C-I	0.694	2.290	2.079	0.727	2.119	2.246
	Model C-II	0.715	2.161	2.203	0.749	2.000	2.380
	Model C-III	0.726	2.028	2.347	0.786	1.830	2.600
	Model C-IV	0.771	1.899	2.506	0.804	1.771	2.687

## Data Availability

Not applicable.
